# Dialysis Patients’ Evaluation of Lung Edema at Home Using a Mobile Phone Tele-Ultrasound Application: A Pilot Study

**DOI:** 10.3390/jcm14020654

**Published:** 2025-01-20

**Authors:** Itamar Ben Shitrit, Aviya Kedmi, Khaled El Haj, Amit Kosto, Ofri Karni, Sharon Einav, Tomer Poleg, Ariel Avraham Hasidim, Noa Bineth, Tomer Gat, Alla Shnaider, Orli Barad, Lior Fuchs

**Affiliations:** 1 Joyce and Irving Goldman Medical School, Faculty of Health Sciences, Ben-Gurion University of the Negev, Beer-Sheva 8410501, Israelliorfuchs@gmail.com (L.F.); 2 Clinical Research Center, Soroka University Medical Center, Faculty of Health Sciences, Ben-Gurion University of the Negev, Beer-Sheva 8410101, Israel; 3 Medical Intensive Care Unit, Soroka University Medical Center, Faculty of Health Sciences, Ben-Gurion University of the Negev, Beer-Sheva 84101, Israel; 4 Maccabi Healthcare System, Sharon Region and Faculty of Medicine, Hebrew University, Jerusalem 9112102, Israel; 5 Department of Pediatrics A, Schneider Children’s Medical Center of Israel, Petah Tikva 94903, Israel; 6 Sackler Faculty of Medicine, Tel Aviv University, Tel Aviv 6997801, Israel; 7 Department of Nephrology, Soroka University Medical Center, Beer-Sheva 8457108, Israel

**Keywords:** home care services, ultrasonography, pulmonary edema, water–electrolyte balance

## Abstract

**Background**: Home rehabilitation improves patient satisfaction and reduces the need for specialist consultations. Hemodialysis is a costly post-ICU service that requires frequent monitoring. Previous studies have demonstrated the feasibility and accuracy of patients self-scanning their lungs with an ultrasound device within the hospital. **Methods**: In this single-center, prospective pilot study, we compared the quality of high-risk elderly patient-generated lung ultrasound images against physician-generated images as our primary outcome. The secondary outcome assessed image quality and B-line quantification between a home device and a gold standard device, when operated by the same clinician. **Results**: We enrolled nine participants (66% male, median age 76 years [IQR 66,79]). Analysis included 402 ultrasound clips (163 patient-generated, 239 physician-generated, and 237 in-clinic gold standard clips). Patient-generated images demonstrated high reliability (92% highly reliable or reliable) and were non-inferior to physician-generated images (*p* < 0.001). There was substantial agreement in B-line classification (Kw = 0.64, 95% CI: 0.46–0.82). The home device, when operated by the same physician, showed non-inferiority to the gold standard device (*p* < 0.001) with substantial B-line classification agreement (Kw = 0.64, 95% CI: 0.51–0.78). **Conclusions**: High-risk elderly patients can successfully generate self-scanned lung ultrasound images comparable to those produced by physicians. These promising results warrant further investigation through larger-scale and long-term studies.

## 1. Introduction

Physician-guided lung ultrasound (LUS) improves quality of life when used to manage pulmonary congestion in high-risk cardiac patients undergoing hemodialysis [1]. As hemodialysis is a costly service, integrating LUS into home-based rehabilitation could enhance patient outcomes and reduce healthcare burdens [2,3]. Home monitoring has been associated with reduced mortality, fewer hospital admissions, and lower overall care costs [2,4,5]. Even among complex cases such as intensive care unit (ICU) survivors, home-based rehabilitation shows promising results. These patients often report greater satisfaction and consult with medical specialists less frequently than those receiving post-admission care [6].

In the last decade, ultrasound devices have developed from a specialized tool for experts to versatile, user-friendly instruments. Moreover, previous research showed that single training sessions of less than 30 min were sufficient to teach a patient to self-scan their lungs effectively [7,8,9]. This development has enabled the application of tele-ultrasound, highlighting its potential for remote use in patients with fluid overload secondary to cardiac or renal etiologies, although its role in primary pulmonary diseases (e.g., obstructive pulmonary disease) is not yet well established [7,8,9,10,11]. When used to remotely monitor COVID-19 lungs, handheld, self-operated ultrasound showcased cost–benefit and high satisfaction rates [7]. Heart failure patients who effectively performed LUS self-scans in a clinical setting were also confident they could conduct such scans at home [9]. Tele-ultrasound should, therefore, be considered for remote monitoring of lung conditions.

In this pilot we studied whether high-risk elderly hemodialysis patients can generate quality LUS at home. The hypotheses were that patients could generate LUS images of B-lines comparable to those generated by physicians (the primary outcome) and that image quality and B-line quantification could be comparable with a home device (Figure S1; Pulsenmore Ltd., Omer, Israel) or a “gold standard” (GS) clinic device (Venue-Go™, GE Healthcare) when operated by the same clinician (the secondary outcome).

## 2. Materials and Methods

For this single-center, prospective, feasibility study (which took place between Aug-Nov 2023), a consecutive sample of hemodialysis patients were enrolled, following institutional review board approval (SOR-0539-20, approval date 3 November 2022) and provision of informed consent. Eligibility criteria included smartphone ownership and ability to operate its text and camera applications, physical ability to self-scan, and intact cognition. All patients had a documented chest CT within the three years before recruitment to confirm the absence of significant pulmonary pathology.

The home device is a portable ultrasound system designed as a cradle for cellular phones, allowing versatile connectivity either through direct phone integration or cable connection (Figure S1). The self-scan process is streamlined for ease of use: a patient connects to the internet, attaches their phone to the device, and is prompted by an application to begin the scan. The application automatically plays a video detailing the scan procedure, guides the patient through the process, and subsequently uploads the videos to a cloud server. The home device’s design and technology are based on a similar device used for pregnancy monitoring, allowing pregnant women to perform follow-ups from home [11].

Training process: Participants were trained to self-scan “Zone 1” of the anterior chest wall using the home device, with additional video tutorials provided for assistance (Figure S2). Each in-clinic training session lasted up to 30 min, consistent with previous literature [7,8,9]. The initial three scans, performed by patients during their hemodialysis sessions, were classified as “training” scans. An ultrasound expert taught basic ultrasonography techniques in these sessions, such as “alignment”, “rotation”, and “tilt”. This training, focused on repetition, was designed to give patients the necessary skills and confidence for precise and independent use of the device at home.

**Figure 1:**
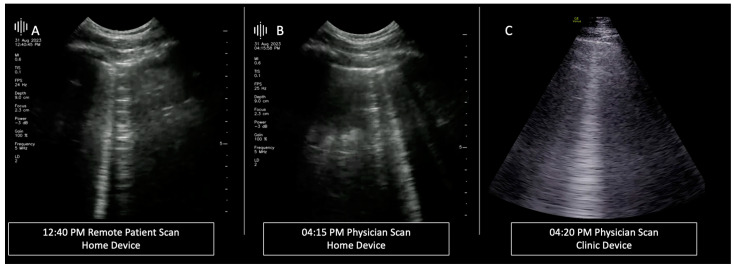
Scan Flow. A sequence is depicted of dialysis ultrasound scans. (**A**) shows a remote patient scan conducted at 12:40 PM using the home device. (**B**) presents a scan performed by a physician at 04:15 PM using the same home device. (**C**) provides a comparison with a physician’s scan at 04:20 PM using the clinic device, showcasing the benchmark quality for lung ultrasound images.

Scan procedure overview: Before each dialysis session, participants performed self-scans at home using the home device (Figure 1) to assess for signs of fluid overload. Upon arriving at the nephrology ward, and within 4 h post-self-scan, a physician, (with more than 3 yr in clinical LUS evaluations), replicated the scans using the home device and the clinic-based GS device before and after dialysis. Post-dialysis, the physician conducted the scans again in the same sequence. All scans, either remote by patients or in-clinic by physicians, were uploaded to a secure cloud server for telemedicine analysis, ensuring efficient and secure data management. A cardiac probe was used for B-line detection in the GS device, and both devices were matched to a fixed gain and a standard 9 cm depth for all measurements to limit potential bias.

Evaluation: An independent ultrasound expert with 10 yr of LUS experience assessed the presence of B-lines and image quality. The reading clinician was blinded to the operator when classifying images (Figure S3). A “highly reliable” score was assigned for images showing two visible ribs, with a clear pleural line between them. A “reliable” score was allocated for images with less clarity or only one visible rib and pleura, while a “non-reliable” score indicated uninterpretable imaging (Figure S3). In the validation of the home device compared to the GS device, B-lines were categorized using a binary system as either pathological or non-pathological, using a threshold of three B-lines.

### Statistical Analysis

Continuous variables with a normal distribution were represented as means and standard deviations (SD). Ordinal or skewed continuous variables were summarized as medians with interquartile ranges (IQR), and categorical variables were represented as counts and percentages. To compare the non-inferiority of image quality produced by physicians versus patients, we used the Wilcoxon signed-rank test with the null hypothesis that the difference between patient and physician image quality is less than or equal to minus one quality level (H0: *μ*patient−*μ*physician ≤ −1). We employed weighted Cohen’s kappa for B-line classification to assess observer agreement across three ordinal levels, allowing us to weigh disagreements differently. We reported the weighted kappa statistics (Kw) with 95% confidence intervals and presented them in an inter-observer kappa agreement matrix, using the expert’s counts from clips captured by the researcher as the “gold standard”. The interpretation of Kw statistics is as follows: Kw = 0 (no better than chance), Kw = 0.01–0.20 (slight agreement), Kw = 0.21–0.40 (fair agreement), Kw = 0.41–0.60 (moderate agreement), Kw = 0.61–0.80 (substantial agreement), Kw = 0.81–0.99 (near-perfect agreement), and Kw = 1.00 (perfect agreement). A mixed model was utilized to account for patient clustering. All statistical tests were two-sided with an alpha level of 0.05, and results were presented with 95% confidence intervals where appropriate. The analyses were conducted using R-Studio software, version 4.4.0.

## 3. Results

**Figure 2:**
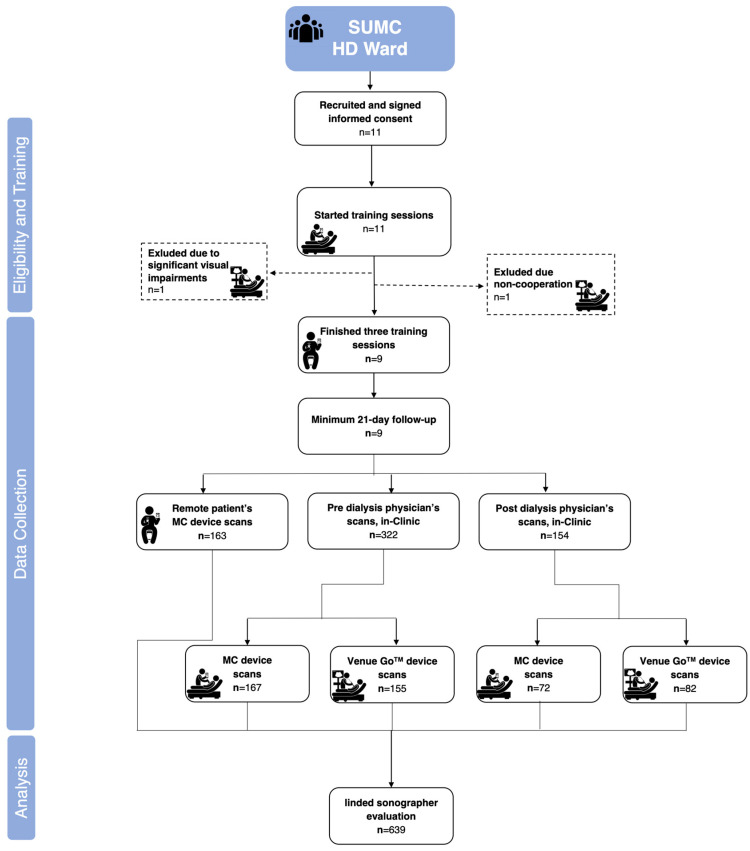
Study flow chart.

A total of 639 ultrasound clips from nine patients were analyzed—402 home ultrasound clips, 163 produced by patients and 239 by physicians, and 237 in-clinic GS device clips (Figure 2), averaging 18 clips per patient (SD 7.6); two participants were excluded during training due to visual impairment and lack of cooperation, yielding the final cohort (n = 9). Participants were mostly males (66%) aged 76 (IQR 66,79) years, undergoing 3 (2,4) weekly dialyses. They had a median body mass index of 29.95 (IQR 21.1, 33.9), a median Charlson’s Comorbidity Index of 11 (IQR 7,13), and were observed for 32.7 days (SD 10.74). No adverse events occurred during the study.

Primary outcome: Most patient-generated scans were highly reliable (72%, 115) or reliable (20%, 33) (Table 1). The quality of images generated by patients (median 2 [1,2]) was non-inferior to those generated by physicians (median 2 [2, 2], *n* = 159, Wilcoxon signed rank test, *p* < 0.001, H0: *μ*patient−*μ*physician ≤ minus one quality level; Table 1). Patient–physician agreement in B-line classification was substantial when using the home device (*n* = 146, weighted kappa = 0.64 [95% CI: 0.46–0.82]). Sex, Charlson Comorbidity Index score, age, BMI, and the number of self-scans were unrelated to B-line classification and scan quality.

Secondary outcome: The quality of images generated by the home device (median 2 [2, 2]) was non-inferior to those generated by the GS device when both were operated by the same physician (median 2 [2, 2], *n* = 219, Wilcoxon signed rank test, *p* < 0.001, H0: *μ*gold standard−*μ*home-device ≤ minus one quality level; Table 1). The degree of agreement between devices in B-line classification was substantial (*n* = 206, weighted kappa = 0.64 [95% CI: 0.51–0.78]).

## 4. Discussion

With appropriate patient selection, some older, chronically ill patients can generate high-quality LUS self-images that are non-inferior to those generated by trained physicians using the same device, thereby enabling remote detection of interstitial changes, which may indicate pulmonary congestion. The accuracy of the home ultrasound device was comparable to that of the GS device for B-line detection and the image quality of the home-device was non-inferior.

The findings of this preliminary study align with prior papers showing that patients can achieve imaging quality that enables remote interpretation when scanning their own lungs at home [7,8,9,11]. Additionally, other studies have highlighted the feasibility of dialysis patients successfully scanning their own lungs and achieving highly accurate results with the support of AI technologies [12]. However, prior studies focused on COVID-19, at-clinic self-scans, and younger patients. Furthermore, although some protocols include lateral views in dialysis patients, our older participants had difficulty positioning themselves to scan these areas, limiting us to the two anterior zones [7,8,9]. This study’s novelty lies in engaging older, chronically ill patients to self-monitor and upload lung ultrasound videos from home to a telemedicine platform, while also attempting to differentiate the impacts of patient and device factors on image quality.

The 2019 KDIGO Controversies Conference emphasized the need for additional research on the relationship between LUS-guided volume management and outcomes [13]. Since then, at least one pilot study has suggested that hemodialysis patients can use artificial intelligence (AI)-assisted LUS in the clinic [12]. In the Patient-PLUS study, heart failure patients, who were also predisposed to pulmonary congestion, effectively performed LUS self-scans in a clinical setting and expressed confidence in their ability to conduct these scans at home [9].

Home-based US monitoring shows potential for ambulatory self-monitoring. Increasing adoption of telehealth services facilitates remote performance and interpretation of imaging, including LUS [8]. Devices have become user-friendly, hand-held, and affordable, while retaining image quality [14]. In addition, AI support enhances diagnostic precision (including B-line counts) [12]. No less importantly, home use of LUS is likely to increase patient autonomy and engagement, which might benefit elderly patients in particular [2,3,7,8,9,11].

This pilot study was conducted by a single medical center with a small number of participants, making patient selection necessary. While most elderly patients are less proficient with smartphone technology than younger patients, it is worth noting that even more complex procedures, such as peritoneal dialysis, can be managed at home with appropriate patient selection [15,16,17]. Logistical challenges, such as patients forgetting to charge their device or leaving it at home, were also observed. This affected follow-up times and the number of scans produced; this is an important lesson in a feasibility study, as it reflects real life.

## 5. Conclusions

Further research is needed to determine whether such practice affects clinical outcomes such as mortality, hospitalization rates, lengths of hospital stay, and quality of life, and to assess the cost–benefit of this practice.

## Figures and Tables

**Table 1:** Comparisons were performed for paired clips, and a paired Wilcoxon signed rank test was used to assess the non-inferiority of patients compared to physicians, as well as home devices versus hospital-based devices, in capturing quality ultrasound images. The null hypothesis posited that the difference between the populations (patients vs. physicians and home device vs. hospital device) was equal to or greater than one level. Overall, a mean of 18 (SD 7.6) clips per patient were assessed (n = 378 home ultrasound clips—159 produced by patients/physicians—and 219 in clinic per device).

Characteristic	Main Outcome		Secondary Outcome	
	Patient N = 159 ^1^	Trained Physician N = 159 ^1^	Home Device N = 219 ^1^	Hospital Device N = 219 ^1^
Quality Category				
Non-Reliable	12 (7.5%)	3 (1.9%)	9 (4.1%)	7 (3.2%)
Reliable	32 (20%)	23 (14%)	33 (15%)	29 (13%)
Highly Reliable	115 (72%)	133 (84%)	177 (81%)	183 (84%)
Descriptive Statistics				
Mean (SD)	1.6 (1)	1.8 (0)	1.8 (1)	1.8 (0)
Median (Q1, Q3)	2 (1, 2)	2 (2, 2)	2 (2, 2)	2 (2, 2)

^1^ n (%).

## Data Availability

The main document and Supplementary Information Files contain the methods and all data that were generated and analyzed in this study. Any raw data not explicitly shown are available from the corresponding author, subject to the approval of the institutional review board (IRB). Any identifiable patient information is protected according to the approved Soroka University Medical Center IRB protocol (IRB# SOR-0539-20).

## Supplementary Materials

The following supporting information can be downloaded at: https://www.mdpi.com/article/10.3390/jcm14020654/s1, Figure S1: The home device, Figure S2: “Zone 1” anterior chest wall position for self-scan, Figure S3: Quality score matrices.
