# Supplementary material for: Dialysis Patients’ Evaluation of Lung Edema at Home Using a Mobile Phone Tele-Ultrasound Application: A Pilot Study
Source: J Clin Med. 2025 Jan 20;14(2):654. doi: 10.3390/jcm14020654 (PMC11765526; doi:10.3390/jcm14020654)
Supplement: Supplementary file 1 [file jcm-14-00654-s001.zip › jcm-3377789-supplementary.pdf]

# Supplementary Materials:

**Dialysis patients' evaluation of lung edema at home using a mobile-phone tele-ultrasound application, a pilot study**

Itamar Ben Shitrit,MPH, [a,b,c], Aviya Kedmi, MD, [a,c], Khaled El Haj, MD[c], Amit Kosto[a,c], Ofri Karni, MD[a], Sharon Einav, MD, MSc[d], Tomer Poleg[a], Ariel Avraham Hasidim, MD, MPH[e,f], Noa Bineth, MD[c], Tomer Gat, MD[a,c], Alla Shnaider, MD[g], Orli Barad[b], Lior Fuchs , MD[a,c]

[a]Joyce and Irving Goldman Medical School, Faculty of Health Sciences, Ben-Gurion University of the Negev, Beer-Sheva, Israel.

[b] Clinical Research Center, Soroka University Medical Center, Faculty of Health Sciences, Ben-Gurion University of the Negev, Beer-Sheva, Israel

[c] Medical Intensive Care Unit, Soroka University Medical Center, Faculty of Health Sciences, Ben-Gurion University of the Negev, Beer-Sheva, Israel.

[d] Maccabi Healthcare System, Sharon Region, and Hebrew University Faculty of Medicine, Jerusalem, Israel

[e] Department of Pediatrics A, Schneider Children's Medical Center of Israel, Petah Tikva, Israel

[f] Sackler Faculty of Medicine, Tel Aviv University, Tel Aviv, Israel

[g] Department of Nephrology, Soroka University Medical Center, Beer-Sheva 8457108, Israel

**Figure S1 – The home device (**Pulsenmore Ltd., Omer, Israel)

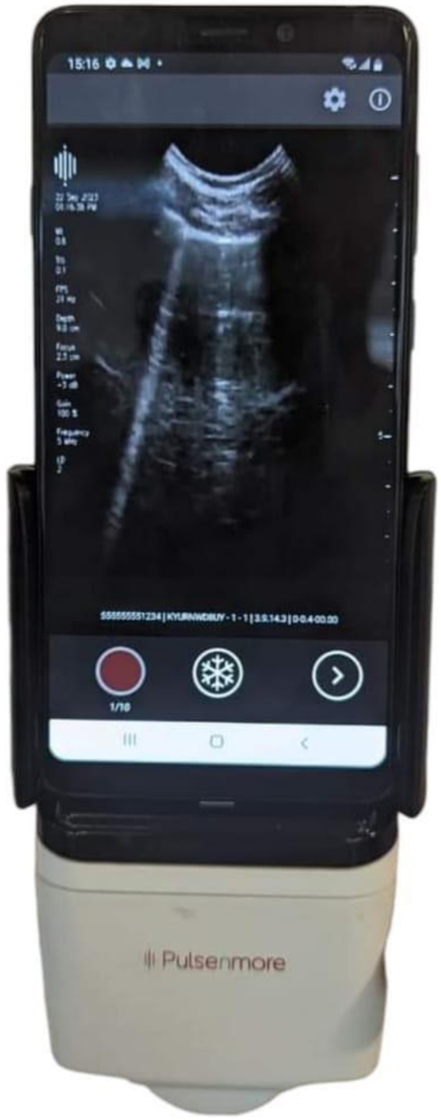

**Figure S2 -** "Zone 1" anterior chest wall position for self-scan

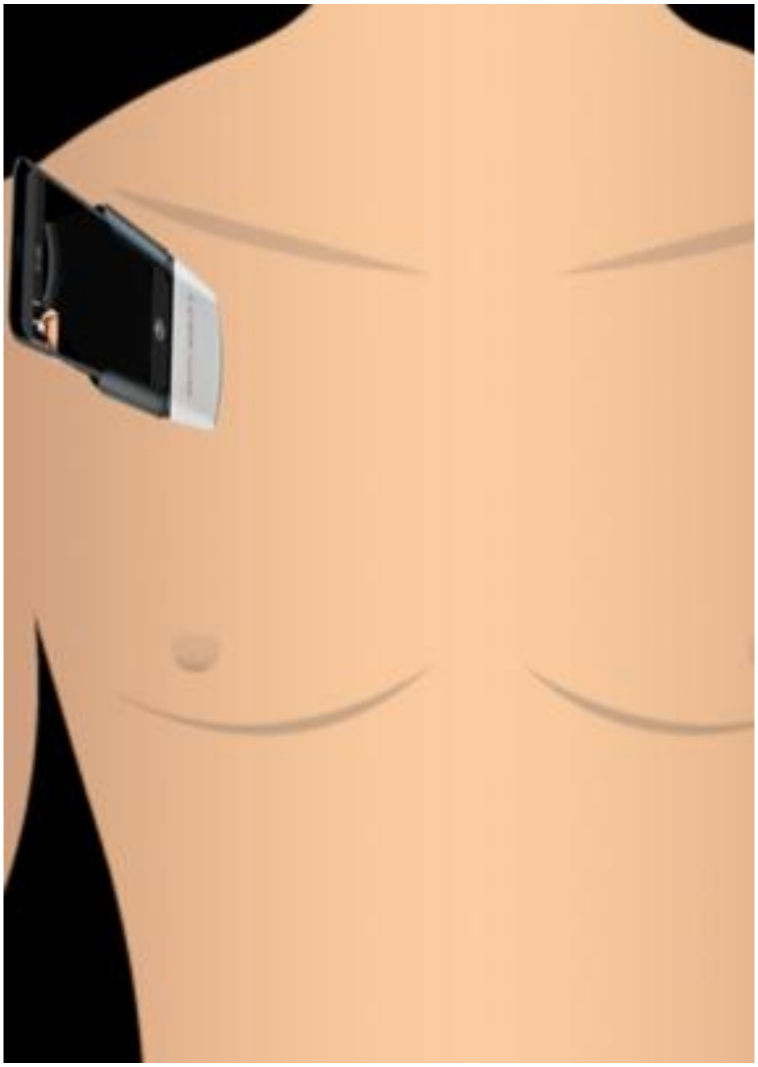

**Figure S3- Quality score matrices**

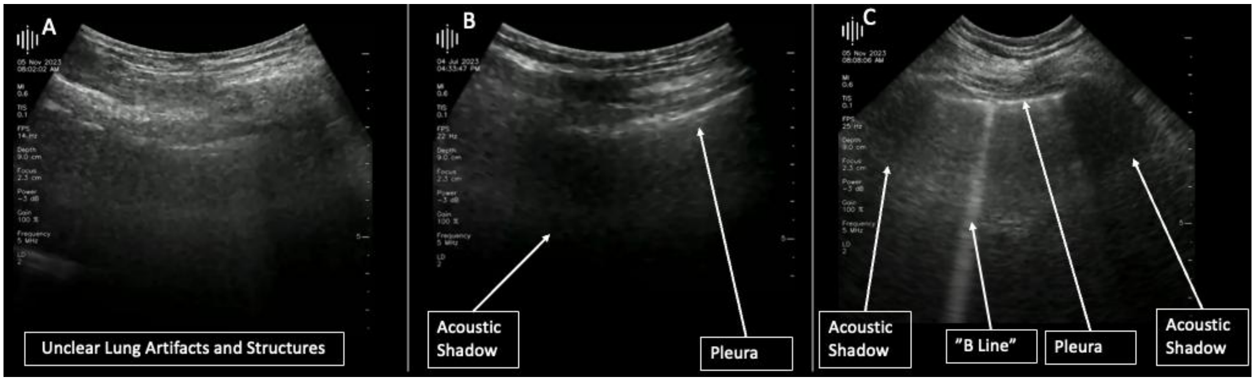


**Figure S3** presents a visual representation of the scoring system used to evaluate lung ultrasound image quality. Panel A is assigned a non-reliable score due to the inability to interpret the image, as indicated by the unclear visualization of the pleura and ribs. Image B is given a reliable score, where the pleura is discernible, but the image clarity of the ribs or the pleura line is suboptimal, or there is only one visible rib with a pleura on both sides. Image C presents a highly reliable score, demonstrating an ideal ultrasound image with two ribs showing characteristic acoustic shadows and a clear, uninterrupted pleural line between them.
